# Integrating functional proteomics and next generation sequencing reveals potential therapeutic targets for Taiwanese breast cancer

**DOI:** 10.1186/s12014-025-09526-8

**Published:** 2025-01-22

**Authors:** Wei-Chi Ku, Chih-Yi Liu, Chi-Jung Huang, Chen-Chung Liao, Yen-Chun Huang, Po-Hsin Kong, Hsieh Chen-Chan, Ling-Ming Tseng, Chi-Cheng Huang

**Affiliations:** 1 School of Medicine, College of Medicine, Fu Jen Catholic University, New Taipei, 242 Taiwan; 2 Division of Pathology, Cathay General Hospital, Taipei, 106 Taiwan; 3 Department of Medical Research, Cathay General Hospital, Taipei, 106 Taiwan; 4 Department of Biochemistry, National Defense Medical Center, Taipei, 114 Taiwan; 5 Cancer and Immunology Research Center, National Yang Ming Chiao Tung University, Taipei, 112 Taiwan; 6 Marker Exploration Corporation, Taipei, 112 Taiwan; 7 Division of Breast Surgery, Department of Surgery, Taipei Veterans General Hospital, Taipei, 112 Taiwan; 8 School of Medicine, College of Medicine, National Yang Ming Chiao Tung University, Taipei, 112 Taiwan; 9 Institute of Epidemiology and Preventive Medicine, National Taiwan University, Taipei, 100 Taiwan

**Keywords:** Functional proteomics, Next-generation sequencing, Whole exome sequencing, Targeted sequencing, Taiwanese breast cancer

## Abstract

Integrating functional proteomics and next-generation sequencing (NGS) offers a comprehensive approach to unraveling the molecular intricacies of breast cancer. This study investigates the functional interplay between genomic alterations and protein expression in Taiwanese breast cancer patients. By analyzing 61 breast cancer samples using tandem mass tag (TMT) labeling and mass spectrometry, coupled with whole-exome sequencing (WES) or targeted sequencing, we identified key genetic mutations and their impact on protein expression. Notably, pathogenic variants in *BRCA1*, *BRCA2*, *PTEN*, and *PIK3CA* were found to be clinically relevant, potentially guiding targeted therapy decisions. Additionally, we discovered trans correlations between specific gene alterations (*FANCA*, *HRAS*, *PIK3CA*, *MAP2K1*, *JAK2*) and the expression of 22 proteins, suggesting potential molecular mechanisms underlying breast cancer development and progression. These findings highlight the power of integrating proteomics and NGS to identify potential therapeutic targets and enhance personalized medicine strategies for Taiwanese breast cancer patients.

**Supplementary Information:**

The online version contains supplementary material available at 10.1186/s12014-025-09526-8.

## Introduction

Breast cancer is the most prevalent cancer among women in Taiwan [1]. While the mortality rate is decreasing in the West, it is increasing in Asian countries, including Taiwan [2]. The average age of onset for breast cancer in Taiwanese women is also younger than in Western women [3]. Despite advancements in breast cancer treatment, there are still unmet clinical needs. These include identifying specific genetic mutations in Taiwanese breast cancer patients to develop more effective targeted therapies [4]. Addressing these unmet needs is crucial to improving the quality of life and survival rates for Taiwanese women with breast cancer.

Proteomics and next generation sequencing (NGS) are powerful tools revolutionizing breast cancer research. Proteomics is the large-scale study of proteins, crucial for understanding the molecular mechanisms of breast cancer. It helps identify biomarkers for early detection, prognosis, and prediction of treatment response [5, 6]. Mass spectrometry (MS)-based proteomics enables comprehensive analysis of protein expression and modifications, shedding light on tumor heterogeneity and potential therapeutic targets. Spatial proteomics, such as imaging mass spectrometry, allows visualizing protein distribution within tissues, providing insights into tumor microenvironment and disease progression [7].

NGS enables rapid sequencing of DNA and RNA, providing valuable information on genetic mutations, gene expression patterns, and tumor evolution [8]. Integrating NGS data with proteomics (proteogenomics) enhances our understanding of how genetic alterations translate into protein changes, driving breast cancer development and progression [9]. Together, proteomics and NGS are accelerating the development of personalized medicine for breast cancer patients. We initiated an approach integrating functional proteomics and NGS, in an effort to understand the molecular alternations underpinning breast cancer development, and to identify potential therapeutics directing to future personalized medicine.

## Materials and methods

### Study population

Pre-operative and treatment-naive core needle biopsy samples stored as formalin-fixed paraffin-embedded (FFPE) pathological archives were used for targeted sequencing and MS analyses [9]. For WES, fresh-frozen samples were collected from tumor and matched adjacent normal tissues during the definite breast cancer surgery. Inclusion criteria were newly diagnosed early breast cancers (stage 0 to III) while exclusion criteria were de novo stage IV disease and insufficient samples. The whole project had been reviewed and approved by the Institutional Review Board with singed informed consent from all participants.

### NGS: whole exome sequencing

The procedure of whole exome sequencing (WES) for both tumor and adjacent normal tissue had been described previously [10, 11]. Exome capture and library preparation was conducted using the Agilent SureSelect XT Reagent Kit and SureSelect XT Human All Exon Version 6 (60 Mb) probe set (Agilent Technologies, Inc.). 1000 ng of genomic DNA was used for library construction. Sequencing was conducted using the Illumina Hiseq4000 with 150 bp paired-end protocol (Illumina, Inc., San Diego, CA, USA). Reads were mapped to human reference genome (hg19) using BWA-MEM [12]. Picard tools were used for sorting BAM files and marking duplicates. GATK Best Practices workflow was used for indel realignment and base quality score recalibration [13]. For variant calling, MuTect2 was used for calling somatic SNVs and indels [14]. Various filters were applied to reduce false positives and variants were annotated using Variant Effect Predictor [15]. The default read depth ranged between 50x to 200x, and 100x was pursued to provide a balance between coverage depth and cost, ensuring that even low-frequency variants were detected reliably.

### NGS: targeted sequencing

The procedure of targeted sequencing had also been described [4]. Tumor DNA was extracted from 10-µm FFPE sections by Welgene Biotech., Taiwan with contaminated RNA removed by RNase. The Agilent HaloPlex Target Enrichment System (Agilent Technologies, USA) was used for library preparation. The circularized target DNA-HaloPlex probe hybrids were captured on streptavidin beads (HaloPlex Magnetic Beads, Agilent Technologies Inc.) and added DNA ligase to close nicks in the hybrids. Target libraries were amplified through 22 cycles of PCR, and all samples were sequenced on Illumina NextSeq500 (Illumina, Inc.) using 150PE protocol. The qualified reads data then went through a genomic alignment against hg19 using BWA to obtain basic sequence information [12]. For target summary, there were 56 genes comprising 991 regions, spanning a region size of 207,948 base pairs.

The procedure of targeted sequencing had also been described [4]. Tumor DNA was extracted from 10-µm sections by Welgene Biotech., Taiwan with contaminated RNA removed by RNase. The Agilent HaloPlex Target Enrichment System was used for library preparation. The circularized target DNA-HaloPlex probe hybrids were captured on streptavidin beads (HaloPlex Magnetic Beads, Agilent Technologies Inc.) and added DNA ligase to close nicks in the hybrids. Target libraries were amplified through 22 cycles of PCR, and all samples were sequenced on Illumina NextSeq500 (Illumina, Inc.) using 150PE protocol. The qualified reads data then went through a genomic alignment against hg19 using BWA to obtain basic sequence information [12]. For target summary, there were 56 genes comprising 991 regions, spanning a region size of 207,948 base pairs. An average 120 mb sequencing amount was generated per sample (average 438x target read depth).

### Protein extraction and functional proteomics

To perform functional proteomics, 10 core needle biopsy FFPE sections, each at least 1 cm in length and 10 μm in thickness, from each patient were used. The tumor percentage was > 80% in each section, as validated by a qualified pathologist (CYL). Proteins from FFPE samples were extracted and trypsin digested as previously described [16]. Digested peptides were desalted and quantified by bicinchoninic acid protein assay (Thermo Fisher Scientific, Waltham, MA, USA). Two microgram peptides from each patient were labeled using the TMTsixplex Isobaric Label Reagents (Thermo Fisher Scientific). All samples were randomly divided into 12 TMT batches. Each batch contained 5 samples and one supermix control, which was prepared by mixing equal amounts of the desalted peptides from 61 cancer samples as control.

### Nanoscale liquid chromatography with tandem mass spectrometry (nanoLC-MS/MS)

NanoLC-MS/MS analyses were conducted with a nanoAcquity UPLC system (Waters, Milford, MA, USA) connected to the Orbitrap Elite hybrid mass spectrometer (Thermo Fisher Scientific). Peptide mixtures in 0.1% formic acid (FA) were loaded onto a C18 BEH column (75 μm ID X 25 cm) packed with 1.7-µm particles at a pore with of 130 Å (Waters, Milford, MA). The peptides were separated using a segmented gradient in 60 min from 5 to 35% solvent B (acetonitrile with 0.1% FA) at a flow rate of 300 nL/min and a column temperature of 35 °C. Solvent A was 0.1% FA in water. The MS was operated in the data-dependent acquisition mode. In brief, survey full scan MS spectra were acquired in the orbitrap (m/z 350–1600) with the resolution set to 60 K at m/z 400 and automatic gain control (AGC) target at 1 million. The 15 most intense ions were sequentially isolated for HCD MS/MS fragmentation and detection in the orbitrap with previously selected ions dynamically excluded for 60 s. For MS/MS, we used a resolution of 15,000, an isolation window of 2 m/z and an AGC target value of 50,000 ions, with the maximal accumulation time of 200 ms. MS/MS fragmentation was performed with normalized collision energy of 35% and an activation time of 0.1 ms. Ions with singly and unrecognized charge state were excluded from MS/MS fragmentation. In this study, two technical repeats for each TMT batch were analyzed.

Protein identification was carried out using the Andromeda search engine, which was incorporated in MaxQuant (v.1.6.12.0) against the SWISSPROT human sequence database (canonical + isoforms) downloaded in Oct 2019 [17, 18]. The enzyme specificity was trypsin with up to two missed cleavages. Cysteine carbamidomethylation was set as a fixed modification. N-acetylation of proteins, oxidation of methionine, and formylation of lysine were set as variable modifications. The minimum peptide length was set to seven amino acids. False discovery rates (FDRs) at the peptide and protein identification levels were fixed at 1%.

### Proteogenomic analysis

Protein quantitation was determined by MaxQuant. PSM-level normalization in MaxQuant was turned on, with PIF filter > 0.75 and “weighted ratio to reference channel” normalization method selected, the latter referred to the reference sample (control group, which was the mixed average of all cancerous samples) in each TMT batch [19]. The intensity from each TMT channel, i.e., each patient, was normalized to the intensity of the supermix control in each TMT batch. The resulting ratios (patient/supermix) were used for further statistical and bioinformatic analyses. To further minimize the impact of technical factors on the quantitation, a median-median-normalization was applied [20].

Due to discrepancy in the genomic regions of interest, only common mutant genes between WES and targeted sequencing were interrogated, namely *AKT1*, *ATM*, *BRCA1*, *BRCA2*, *EGFR*, *FGFR3*, *FNACA*, *JAK2*, *MAP2K2*, *MET*, *NOTCH1*, *RET*, *SMO* and *TP53*. We only consider somatic mutations from WES as no germline controls from targeted sequencing. Both cis and trans correlations between genomic alterations and protein expression, with Pearson correlation coefficients and accompanied P-value reported. The Bonferroni correction was used to address the issue of multiple comparisons and reduce type I error (false positive) with an induced alpha-level of 5 × 10^− 4^ [21]. All variants, regardless of functionality, were used for proteogenomic correlation analysis while oncogenicity of reported variants was ascertained by the OncoKB database [22, 23].

## Results

### Study population

The study population contained 61 Taiwanese breast cancers with functional proteomics data. Table 1 details TMT batch, controls and immunohistochemistry (IHC) subtypes based on hormone receptor (HR) and human epidermal growth factor receptor II (HER2) status. There were 29 h+/HER2-, 16 h+/HER2+, 9 h-/HER2 + and 7 h-/HER2- cases.

### NGS experiments

**Fig. 1:**
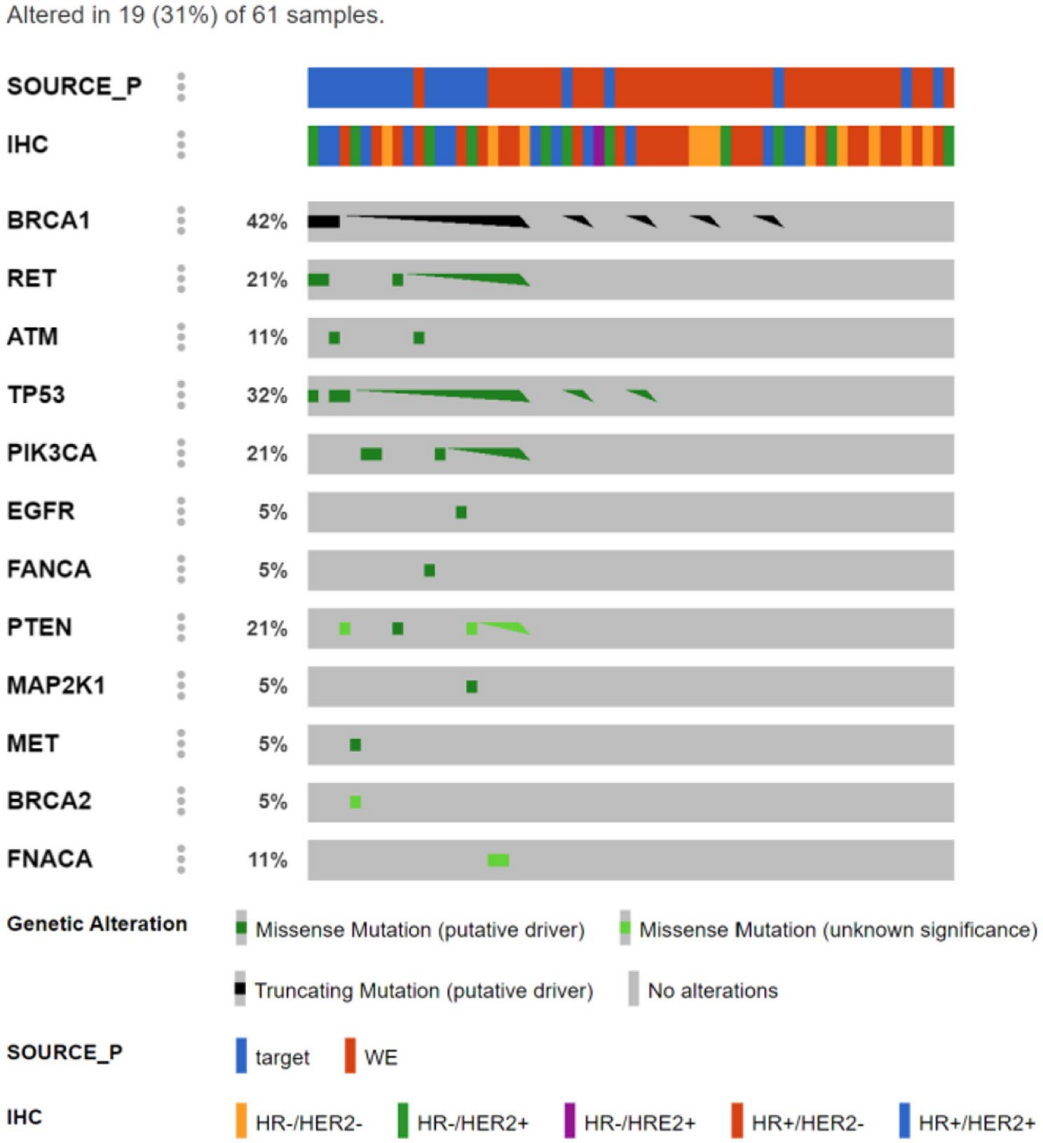
Mutational landscape of Taiwanese breast cancer underwent next generation sequencing

Among 61 Taiwanese breast cancers, 40 underwent WES and the remaining underwent targeted sequencing. Figure 1 shows mutational landscape of common altered genes between both NGS platforms. Pathogenic variants were observed in 19 (31%) of the study cohort, with *BRCA1* as the most prevalence (13%) and *TP53* (10%). Supplementary Table 1 details genomic alterations of Taiwanese breast cancers, with and without functional pathogenicity.

**Table 1:** Tandem mass tag (TMT) and immunohistochemistry (IHC) subtypes of the study population

Batch	TMT	Sample ID	IHC subtype	Batch	TMT	Sample ID	IHC subtype
1	126	SA33	HR+/HER2-	7	126	SA23	HR+/HER2-
1	127	SA28	HR-/HER2-	7	127	SA05	HR-/HER2+
1	128	SA10	HR+/HER2-	7	128	Control7	Control
1	129	SA36	HR+/HER2+	7	129	SA17	HR+/HER2-
1	130	Control1	Control	7	130	SA34	HR+/HER2-
1	131	SA06	HR+/HER2+	7	131	SA44	HR+/HER2+
2	126	SA48	HR-/HER2-	8	126	SA18	HR+/HER2-
2	127	Control2	Control	8	127	SA35	HR-/HER2+
2	128	SA30	HR-/HER2+	8	128	SA11	HR+/HER2+
2	129	SA27	HR+/HER2-	8	129	SA45	HR-/HER2-
2	130	SA13	HR-/HRE2+	8	130	Control8	Control
2	131	SA14	HR-/HER2+	8	131	SA21	HR+/HER2-
3	126	SA03	HR-/HER2-	9	126	SA20	HR+/HER2-
3	127	SA29	HR+/HER2+	9	127	Control9	Control
3	128	SA16	HR+/HER2+	9	128	SA04	HR+/HER2+
3	129	Control3	Control	9	129	SA32	HR+/HER2-
3	130	SA37	HR+/HER2-	9	130	SA15	HR+/HER2-
3	131	SA38	HR-/HER2+	9	131	SA01	HR+/HER2+
4	126	Control4	Control	10	126	SA59	HR-/HER2-
4	127	SA22	HR+/HER2-	10	127	SA55	HR-/HER2-
4	128	SA02	HR+/HER2-	10	128	Control10	Control
4	129	SA07	HR-/HER2-	10	129	SA61	HR-/HER2+
4	130	SA42	HR-/HER2+	10	130	SA39	HR-/HER2+
4	131	SA09	HR-/HER2+	10	131	SA52	HR-/HER2-
5	126	SA43	HR+/HER2+	11	126	SA57	HR+/HER2-
5	127	Control5	Control	11	127	Control11	Control
5	128	SA40	HR-/HER2+	11	128	SA53	HR+/HER2-
5	129	SA47	HR-/HER2+	11	129	SA56	HR+/HER2+
5	130	SA41	HR+/HER2-	11	130	SA31	HR+/HER2+
5	131	SA19	HR+/HER2-	11	131	SA51	HR-/HER2-
6	126	SA12	HR+/HER2-	12	126	SA54	HR+/HER2-
6	127	SA46	HR+/HER2-	12	127	SA60	HR+/HER2-
6	128	SA24	HR-/HER2-	12	128	SA08	HR+/HER2+
6	129	SA26	HR-/HER2-	12	129	SA58	HR+/HER2-
6	130	SA49	HR+/HER2-	12	130	SA50	HR+/HER2-
6	131	Control6	Control	12	131	Control12	Control

(HR: hormone receptor, HER2: human epidermal growth factor receptor II)

### Proteogenomic analyses

**Fig. 2:**
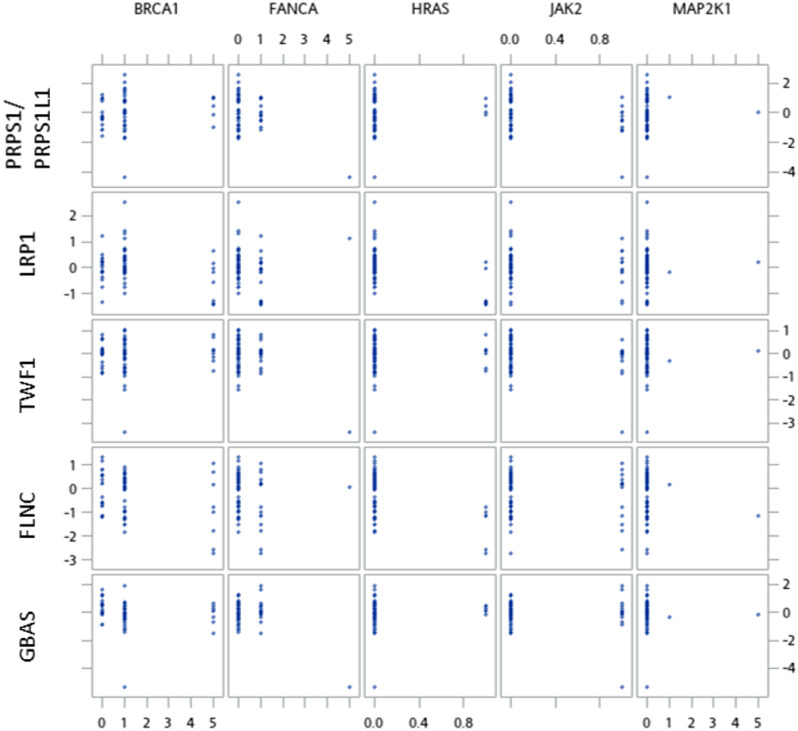
Scatter plot matrix of proteogenomic analyses among 61 Taiwanese breast cancers

No cis correlation was observed in this study while trans correlations between genomic alteration and protein expression were pronounced after controlling for false discovery (Table 2; Fig. 2). Negative correlations were noted between *FANCA* alterations and protein expression of GBAS, SFXN3, TWF1, CPPED1, EIF3L, COPE, PRPS1/PRPS1L1, PHB; *HRAS* alterations and NEB, FLNC, LRP1 protein expression; *PIK3CA* alterations and TPM3, ANXA6, EEF1D, SSR3, HMGB1, HMGB1P1 protein expression; *MAP2K1* alteration and PPP1CC expression while positive correlations between *PIK3CA* alterations and SAMM50, RANGAP1 expression as well as *JAK2* alteration and ITGA11 expression.

**Fig. 3:**
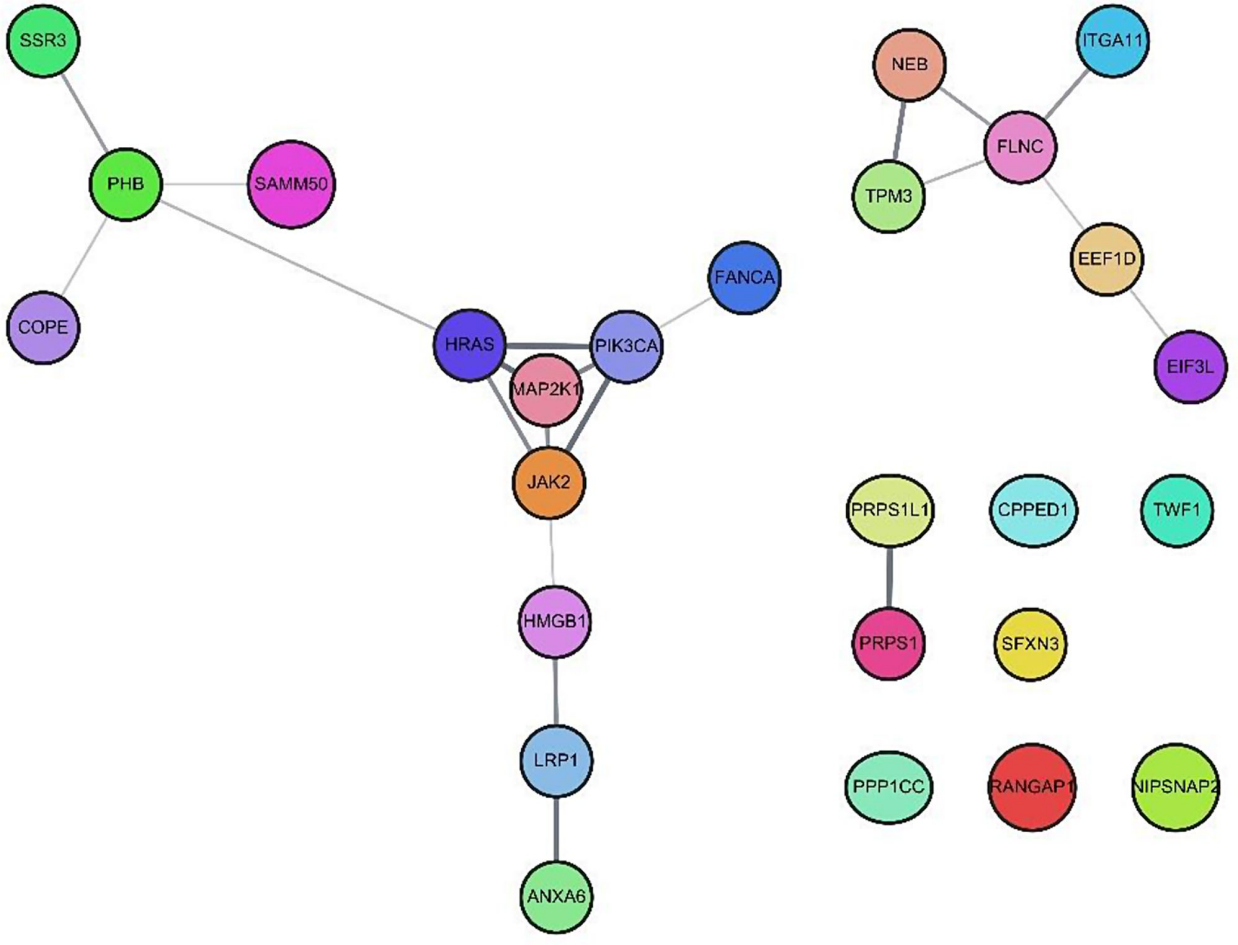
The STRING network view of genomic alterations and impacted protein expressions among 61 Taiwanese breast cancers

To further understand the molecular interplay between genomic alterations and protein expression, we put all 27 candidates, including 5 altered genes (*FANCA*, *HRAS PIK3CA*, *MAP2K1*, *JAK2*) and 22 impacted proteins into the STRING database using default setting, and Fig. 3 displays network view summering the predicted associations for these proteogenomic candidates [24]. The protein-protein interaction (PPI) enrichment P-value was 0.00126, indicating input proteins have more interactions among themselves than what would be expected for a random set of proteins of the same size and degree distribution drawn from the genome. Such an enrichment indicates that the proteins are at least partially biologically connected, as a group. Functional enrichments with more than 2 of strength in terms of log_10_(observed / expected) included ribose phosphate diphosphokinase activity (molecular function), ribose phosphate diphosphokinase complex (cellular component), 5-Phosphoribose 1-diphosphate biosynthesis, MAPK3 (ERK1) activation, signaling by FGFR4 in disease, signaling by PDGFRA extracellular domain mutants, signaling by PDGFRA transmembrane, juxtamembrane and kinase domain mutants (Reactome), PDGFR-beta pathway (WikiPathways) and N-terminal domain of ribose phosphate pyrophosphokinase (protein domains, SMART) [25–26]. Supplementary Table 1 details enrichment analysis results.

**Table 2:** Correlations between genomic alteration and protein expression among 61 Taiwanese breast cancers

Gene	Protein	Correlation	*P*-value
FANCA	GBAS	-0.5114828	0.0001
FANCA	SFXN3	-0.5107265	0.0001
HRAS	NEB	-0.5698695	0.0001
PIK3CA	SAMM50	0.57097525	0.0001
PIK3CA	TPM3	-0.4831809	0.0001
PIK3CA	ANXA6	-0.4811268	0.0001
PIK3CA	EEF1D	-0.483507	0.0001
FANCA	TWF1	-0.470296	0.0002
FANCA	CPPED1	-0.4775763	0.0002
HRAS	FLNC	-0.4880356	0.0002
MAP2K1	PPP1CC	-0.5575056	0.0002
PIK3CA	SSR3	-0.5012579	0.0002
FANCA	EIF3L	-0.477688	0.0003
FANCA	COPE	-0.4861374	0.0003
HRAS	LRP1	-0.4535422	0.0003
JAK2	ITGA11	0.5481589	0.0003
PIK3CA	RANGAP1	0.45661621	0.0003
FANCA	PRPS1;PRPS1L1	-0.4879296	0.0004
FANCA	PHB	-0.4476773	0.0004
PIK3CA	HMGB1;HMGB1P1	-0.4505585	0.0004

## Discussion

Functional proteomics is a powerful approach for studying breast cancer that focuses on analyzing protein expression, interactions, and functions. Key aspects include identifying biomarker panels for prognosis and treatment prediction, profiling protein expression in large cohorts of breast cancer specimens to link proteomic data with clinical outcomes and exploring the complexity of breast cancer proteomes to identify potential therapeutic targets and improve patient stratification [27–29]. Functional proteomics has significant potential to enhance breast cancer management by providing insights into disease mechanisms, identifying novel biomarkers, and guiding personalized treatment strategies.

NGS, coupled with matched treatment, has shown to enhance clinical outcomes [30].

NGS testing allows for molecular-guided treatment decisions, offering new targeted therapy options. With identification of actionable mutations, NGS helps detect mutations that can be targeted with specific therapies. Combining NGS with functional proteomics offers several benefits for breast cancer research and clinical applications such as comprehensive molecular profiling, enhanced heterogeneity analysis as both genomic and proteomic levels are deciphered.

In current study, we took advantage of 61 Taiwanese breast cancers who underwent functional proteomics coupled with either WES or targeted sequencing. A couple of pathogenic mutations were identified and were clinically relevant: namely *BRCA1/2*, *PTEN* and *PIK3CA*. Both *BRCA1* and *BRCA2* are well-known tumor suppressors while Poly (ADP-ribose) polymerase (PARP) inhibitors are FDA-approved for patients with germline *BRCA1/2* mutant ovarian and breast cancers [31]. *PTEN* is a tumor suppressor that is one of the most frequently mutated genes in human cancer, and the pan-AKT kinase inhibitor capivasertib in combination with the selective estrogen receptor degrader (SERD) fulvestrant is FDA-approved for the treatment of patients with *PTEN*-mutant HR+/HER2- metastatic breast cancer [32]. *PIK3CA*, the catalytic subunit of PI3-kinase, is frequently mutated in a diverse range of cancers including breast, endometrial and cervical cancers, and the alpha-isoform selective PI(3)-kinase inhibitor alpelisib and the pan-AKT kinase inhibitor capivasertib, each in combination with fulvestrant, are FDA-approved for the treatment of patients with *PIK3CA* mutant ER+/HER2- metastatic breast cancer [32, 33]. Other pathogenic mutations are currently not actionable for breast cancer treatment.

Five genes, including *FANCA*, *HRAS*, *PIK3CA*, *MAP2K1* and *JAK2*, were shown to impact distant (trans) protein expressions, with most being negatively correlated. *FANCA* is a tumor suppressor and DNA repair protein with germline mutations of *FANCA* associated with the cancer predisposition syndrome Fanconi Anemia. *HRAS*, a GTPase, is commonly mutant in head and neck, thyroid, and bladder cancer. The Ras proto-oncogene family (*HRAS*, *NRAS* and *KRAS*) is the upstream of pro-proliferative and anti-apoptotic signal transduction pathways, including the mitogen activated protein kinase (MAPK) and PI3 kinase (PI3K) pathways [34]. MAP2K1 (MEK1), which is infrequently mutated in melanoma, colon and lung cancer, is involved in the RAS/MAPK signaling pathway, influencing various cellular processes such as growth, proliferation, and survival [35]. *JAK2*, a non-receptor tyrosine kinase, is commonly mutant in hematologic malignancies such as myeloproliferative neoplasm [36]. Except *PIK3CA*, none of these impacting genes were breast cancer actionable.

Twenty-two proteins were impacted by 5 altered genes, with most being negatively. GBAS (NIPSNAP2) a positive regulator of L-type calcium channels, belonging to the NipSnap family. SFXN3 is a mitochondrial protein that functions as a serine transporter, facilitating the transport of serine into the mitochondria, and is also involved in iron transport [37]. NEB is involved in maintaining the structural integrity of sarcomeres and the membrane system associated with the myofibrils. SAMM50 maintains the structure of mitochondrial cristae and the proper assembly of the mitochondrial respiratory chain complexes. *TPM3* gene encodes the slow muscle alpha (α)-tropomyosin protein, which belongs to the tropomyosin family, a group of actin-binding proteins [38]. ANXA6 is associated with CD21 and regulates the release of Ca^2+^ from intracellular stores [39]. As a subunit of elongation factor-1 complex (EEF1), EEF1D is essential for protein synthesis, delivering aminoacyl tRNAs to ribosome, and short isoforms through alternative splicing may be pathogenic [40]. *TWF1* gene encodes an actin monomer-binding protein, which is essential for cytoskeletal remodeling, myogenic differentiation and cancer progression [41]. CPPED1 belongs to the calcineurin-like phosphoesterase domain family and has protein phosphatase activity, specifically targeting serine and threonine residues. *FLNC* gene encodes Filamin-C, a protein that plays a key role in the structure and function of muscles. Protein phosphatase 1 catalytic subunit gamma (PP1-gamma), encoded by PPP1CC, opposes the action of kinases and phosphorylases and is involved in signal transduction. *SSR3* gene encodes the gamma subunit of the signal sequence receptor (SSR), which is a protein complex involved in the recognition and targeting of proteins to the endoplasmic reticulum for further processing and secretion. Eukaryotic translation initiation factor 3 (eIF-3) complex is encoded by *EIF3L*, while this complex is essential for protein synthesis. *COPE* gene encodes an a subunit of the coatomer protein complex, which plays a vital role in intracellular protein transport, specifically in the retrograde transport of proteins from the Golgi apparatus back to the endoplasmic reticulum. Low-density lipoprotein receptor-related protein 1, encoded by *LRP1* gene, is a large endocytic cell surface receptor involved in cell adhesion, signaling, trafficking and degradation of ligands. *ITGA11* gene encodes integrin subunit alpha 11, a protein that forms part of the integrin, which is a cell adhesion molecule. RANGAP1 associates with the nuclear pore complex and acts as a GTPase activator for Ran, a small GTPase that plays a crucial role in nucleo-cytoplasmic transport converts GTP-bound Ran to GDP-bound Ran, which is essential for the directionality and fidelity of nuclear transport [42]. Phosphoribosyl pyrophosphate synthetase 1 (PRPP synthetase 1), encoded by *PRPS1* gene, plays a crucial role in the production of phosphoribosyl pyrophosphate (PRPP), a molecule involved in the synthesis of purine and pyrimidine nucleotides, which are the building blocks of DNA and RNA. Prohibitin protein (encoded by *PHB*) plays a role in cellular senescence and tumor suppression in humans [43]. High Mobility Group Box 1 is a protein encoded by *HMGB1* gene and is a non-histone chromosomal protein, functioning in DNA binding and chromatin architecture, inflammatory response, and autophagy. A diverse set of proteins were correlated with altered genes, and most of which were negatively correlated. Despite this, we could still categorize these proteins into cellular processes and signaling (SFXN3, SAMM50, EEF1D, TWF1, CPPED1, PPP1CC, SSR3, EIF3L, COPE, RANGAP1, PRPS1, PHB, HMGB1), muscle and structure proteins (NEB, TPM3, FLNC, ITGA11) and others (GBAS, ANXA6, LRP1, PRPS1L1, HMGB1P1).

It deserves notice that several studies evaluating the association between PHB expression and breast cancer [44, 45]. In our study, altered *FANCA* was associated with a reduced PHB protein expression. TWF1 has also been linked to breast cancer progression, while our study indicated that *FANCA* alterations negatively impacted TWF1 expression [46] . SFXN3 and ANXA6 had also been reported to be over-expressed in breast cancer [37, 39]. To ascertain that our findings were not spurious, public domain GDC TCGA Breast Cancer (BRCA) database was consulted and elevated *TWF1* mRNA expression did show a trend toward poor overall survival (Supplementary Fig. 1) while *PIK3CA* alterations did impact *ANXA6* mRNA expression (Supplementary Fig. 2) [47].

Targeting the identified protein alterations for therapeutic intervention has challenges but holds promise [48, 49]. Proteins are often complex structures, making them difficult for drugs to target effectively. However, the study provides insights for future research avenues such as targeting upstream pathways and interacted proteins. By understanding the affected pathways due to the genetic alterations (e.g., FANCA mutations), scientists can develop drugs that target these pathways indirectly, affecting protein function. In addition, identified correlations between proteins (e.g., PIK3CA and SAMM50) could be starting points to explore potential drugs that disrupt these interactions. Overall, this research paves the way for future development of targeted therapies in breast cancer, but more research is needed to translate these findings into clinical applications.

The use of different sequencing platforms deserves further discussion. First, targeted panels focus on pre-selected genes, potentially missing variants in unexplored areas, contrasting to the full coverage of WES. Second, targeted panels may not cover all exons within the targeted genes, leading to potential missed variants [50, 51]. On the other hand, read depth can introduce bias between WES and targeted sequencing. Targeted sequencing focuses on specific regions, allowing for higher read depth in those areas compared to WES which covers the entire exome. Lower read depth in WES might lead to missing true variants, especially for those with a much lower variant allele frequency (VAF) [52]. Conversely, very high depth in targeted sequencing can introduce noise. Consideration of read depth is crucial for data analysis and variant identification in both WES and targeted sequencing studies. The higher read depth of targeted sequencing may be associated with more accurate variant calling, especially for rare variants.

There were some limitations of the study. First, limited sample size might compromise the generalizability and external validation of the findings, and further validation with a larger cohort would strengthen the study’s conclusions. Second, NGS was performed with either WES or targeted sequencing due to paucity of resources, and the discrepancy in sequencing technologies and genomic regions inevitable introduced bias. More samples with uniform sequencing technology will bring further inside for the interplay between genomic and proteomic changes. Besides, only common and altered genes between both platforms were investigated, and the reported mutational landscape and frequency might be skewed to the interrogated genes and not necessarily similar to studies with larger panels [53]. We only consider somatic mutations from WES as no germline controls from targeted sequencing, consequently, it is not possible to differentiate germline from somatic mutations, especially for targeted sequencing, which might be an issue for PARP inhibition. Third, distinct molecular aberrations might exist across distinct breast cancer subtypes, future studies should focus on subtype-specific proteogenomic details with more samples assayed. Incorporating external validation cohorts with larger sample sizes is a crucial to confirm generalizability and reduce the chance of false positives.

In conclusion, through combining NGS and functional proteomics, a more holistic view of breast cancer could be expected for improving diagnosis, prognosis, and treatment outcomes. A larger cohort can reveal if the proteomic correlations hold true in a broader breast cancer population, and increase the statistical power to detect true correlations. Validation in external cohorts would significantly strengthen the confidence in the observed relationships between genetic alterations and protein expression.

## Electronic supplementary material

Below is the link to the electronic supplementary material.

Supplementary Material 1

Supplementary Material 2

## Data Availability

All nanoLC-MS/MS raw files and MaxQuant-generated result data have been deposited to the ProteomeXchange Consortium (http://proteomecentral.proteomexchange.org) via the PRIDE partner repository with the dataset identifier PXD038543.
