# Supplementary material for: Integrating functional proteomics and next generation sequencing reveals potential therapeutic targets for Taiwanese breast cancer
Source: Clin Proteomics. 2025 Jan 22;22:4. doi: 10.1186/s12014-025-09526-8 (PMC11753163; doi:10.1186/s12014-025-09526-8)
Supplement: Supplementary file 1 — Supplementary Material 1 [file 12014_2025_9526_MOESM1_ESM.docx]

**Supplementary Figures**

**
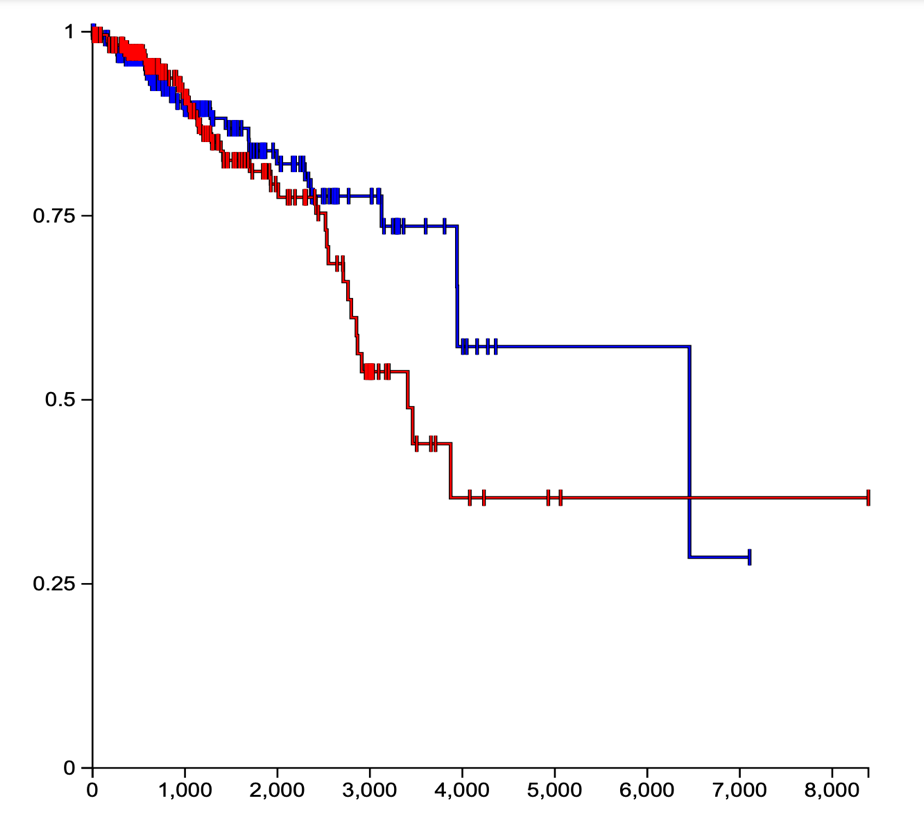
**

**Supplementary Fig. 1.** Elevated *TWF1* mRNA expression did show a trend toward poor overall survival between the first and the last quartiles (log-rank test, P=0.20).

**
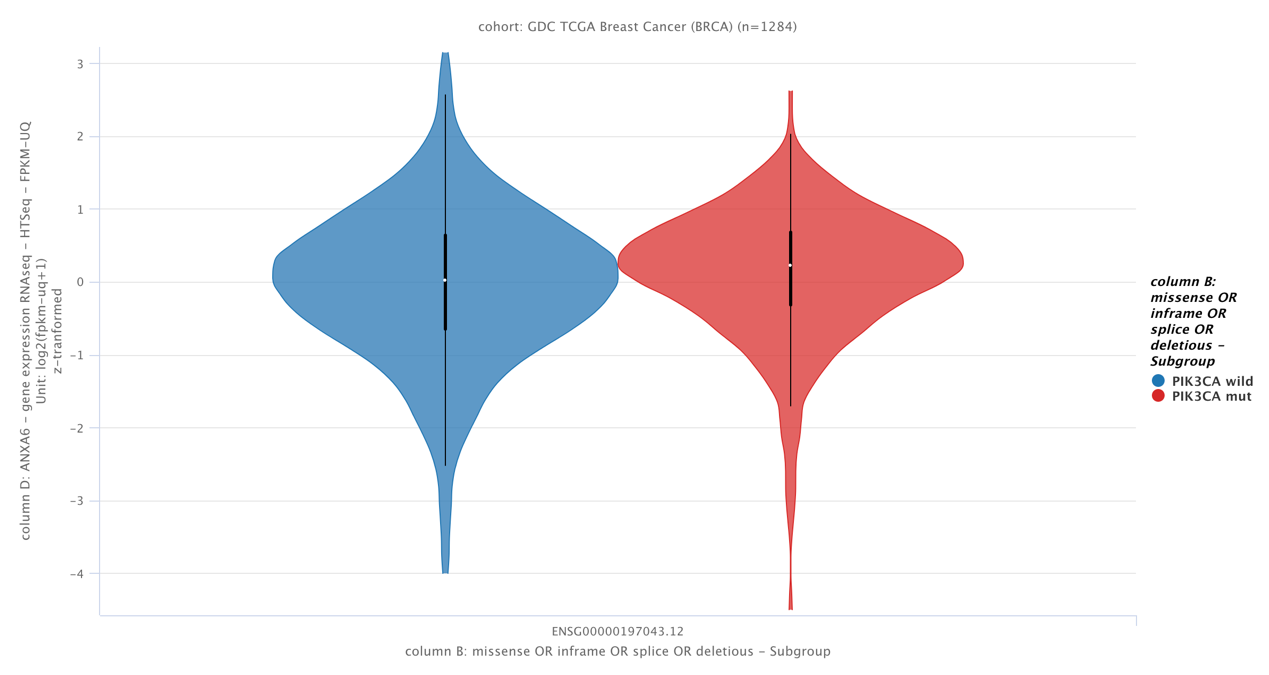
Supplementary Fig 2.** *ANXA6* mRNA was differentially expressed between *PIK3CA* mutant and wild breast cancers (Welch's t-test, P = 0.01).
